# Carotenoids from Different Pumpkin Varieties Exert a Cytotoxic Effect on Human Neuroblastoma SH-SY5Y Cells

**DOI:** 10.3390/nu16173043

**Published:** 2024-09-09

**Authors:** Nicola Pinna, Federica Ianni, Carmela Conte, Michela Codini, Raffaella di Vito, Stefania Urbani, Roberto Selvaggini, Lina Cossignani, Francesca Blasi

**Affiliations:** 1 Department of Pharmaceutical Sciences, University of Perugia, 06126 Perugia, Italy; nicola.pinna@dottorandi.unipg.it (N.P.); federica.ianni@unipg.it (F.I.); carmela.conte@unipg.it (C.C.); michela.codini@unipg.it (M.C.); raffaella.divito@outlook.it (R.d.V.); francesca.blasi@unipg.it (F.B.); 2 Department of Agricultural, Food and Environmental Sciences, University of Perugia, 06126 Perugia, Italy; stefania.urbani99@gmail.com (S.U.); roberto.selvaggini@unipg.it (R.S.)

**Keywords:** *Cucurbita*, bioactives, functional food, vegetable extracts, neurotoxicity, UHPLC-MS/MS

## Abstract

Plants, including pumpkins (*Cucurbita* spp.), are an interesting source of nutrients and bioactives with various health benefits. In this research, carotenoid extracts obtained from the pulp of eight pumpkin varieties, belonging to the *C. moschata* and *C. maxima* species, were tested for cytotoxicity on SH-SY5Y neuroblastoma cells. The results showed that pumpkin bioactives exert a cytotoxic action against the tested cells, in particular Butternut extract at a 100 μM (53.69 μg/mL) concentration after 24 h of treatment and Mantovana extract at 50 μM (26.84 μg/mL) after 48 h. Moreover, the carotenoid extracts also showed interesting in vitro antioxidant activity, evaluated by ABTS and ORAC assays. To fully characterize the qualitative and quantitative profile of carotenoids in the tested extracts, a high-performance chromatographic technique was performed, revealing that pumpkin pulp carotenoids were mainly represented by β-carotene, mono- and di-esterified hydroxy- and epoxy-carotenoids. Moreover, the carotenoid dataset was also useful for discriminating samples from two different species. In conclusion, the results of the present study highlight the potential anti-cancer activity of pumpkin carotenoid extracts and the possibility of using them as chemotherapeutic adjuvants.

## 1. Introduction

Around the world, great progress has been made in improving human health, especially in the past century [1]. Plant foods are an interesting source of carotenoids, which are bioactive compounds for humans. Epidemiological evidence indicates that health benefits (i.e., reduction of risk of developing certain cancers, cardiovascular diseases, and eye disorders) could be expected from carotenoid daily intake; therefore, the interest in these bioactives has expanded considerably in food and pharmaceutical fields [2]. Among the functional food rich in carotenoids, pumpkin (*Cucurbita* spp.) should be mentioned, as one of the most consumed vegetables belonging to the Cucurbitaceae family in the world. The whole plant is edible, although the pulp and seeds are especially valuable for the food industry; in fact, pumpkin pulp is mostly used for preparing dishes, even if a new trend is to use its powder as a natural pigment [3]. Furthermore, as there is a growing interest in extracting novel bioactives from foods and agri-food waste by applying innovative procedures (i.e., ultrasound- and microwave-assisted extraction -UAE and MAE-), pumpkin and its waste are valuable for their carotenoids [4,5].

Taking into account different pumpkin varieties, the first works concerned mostly the chemical properties of pumpkin seeds and oils [6,7], while more recent papers are about the phytochemicals and health properties of both the pulp and byproducts [8,9,10]. As an example, Kulczyński and Gramza-Michałowska (2019) studied the content of bioactives (carotenoids, phenolic acids, flavonols, minerals, and vitamins) of the pulp of numerous pumpkin varieties belonging to the *C. pepo* and *C. moschata* species [8]. More recently, Kostecka-Gugała et al. (2020) evaluated the antioxidant capacity of Cucurbita fruits of numerous cultivars belonging to four species, grown in central Europe [9]. The effect of variety and farming type on the nutritional characterization of butternut squash was also studied [11]. It is known that antioxidants, including carotenoids, may provide a protective effect by modulating biochemical processes involved in cell proliferation and apoptosis. Moreover, it is known that oxidative stress has been linked to the onset and progression of different neoplasia; in fact, antioxidants have been shown to counteract or prevent the onset of several malignancies, including brain tumors [12]. Among antioxidants, carotenoids are considered useful in the framework of predictive, preventive and personalized (3P) medicine against cancer development and progression [13]. Several studies have shown that pumpkin extracts can inhibit the proliferation of various cancer cell lines [14]. A pumpkin carotenoid-rich extract delayed a human chronic lymphocytic leukemia cell proliferation by modulating autophagic flux [15]. Carotenoids like lutein and zeaxanthin are also believed to support cognitive function and protect neurons from damage due to their ability to cross the blood-brain barrier and accumulate in the brain [16]. Accordingly, a characterization of carotenoids’ biological activity against several brain cancer cell lines has been reported. Murakoshi et al. (1989) demonstrated that α-carotene inhibits the GOTO neuroblastoma human cell line by promoting G0/G1 cell cycle arrest and increasing N-MYC expression [17]. Similarly, β-carotene has shown anti-metastatic potential in human SK-N-BE(2)C cells in vitro and in vivo [18].

In this paper, carotenoid extracts from eight varieties of pumpkin belonging to the *C. moschata* and *C. maxima* species, obtained by UAE as reported in a previous paper [19], were tested for cytotoxicity on SH-SY5Y neuroblastoma cells. Moreover, the extracts’ in vitro antioxidant activity by spectrophotometric assays and the carotenoid profiling by liquid chromatography-diode array detection (HPLC-DAD) were also studied.

## 2. Materials and Methods

### 2.1. Plant Materials

Pumpkins of eight different varieties belonging to the *C. moschata* (Butternut, Lunga di Napoli, Moscata di Provenza, and Violina rugosa) and *C. maxima* (Delica, Delica vanity, Hokkaido, and Mantovana) species were harvested in October 2021 in Umbria (central Italy). The samples of pumpkin pulp, obtained as reported in a previous paper [19], were stored in amber glass containers, until carotenoid isolation.

### 2.2. Reagents

(±)-6-hydroxy-2,5,7,8-tetramethylchromane-2-carboxylic acid (Trolox), 2,2’-azino-bis(3-ethylbenzothiazoline-6-sulphonic acid) diammonium salt (ABTS), and fluorescein sodium salt were purchased from Sigma-Aldrich (Milan, Italy). Isopropanol, methanol, methyl tert-butyl ether (MTBE), and water of a chromatographic grade were obtained from Carlo Erba Reagents (Milan, Italy). Lutein (≥92%) was purchased from Extrasynthese (Genay, France) and β-carotene (>97.0%) from the Tokyo Chemical Industry Company (Toshima, Kitaku, Tokyo, Japan). Antibiotics (penicillin and streptomycin), fetal bovine serum (FBS), Dulbecco’s modified eagle medium (DMEM) high glucose, trypsin-EDTA, L-glutamine, phosphate-buffered saline, and pH 7.4 (PBS) were obtained from Euroclone S.p.A. (Milan, Italy). Acridine orange (AO), 6,4′-diamidino-2-phenylindole (DAPI), and NC-Slide A8 were purchased from ChemoMetec A/S (Allerød, Denmark).

### 2.3. Moisture Content and Color of Pumpkin Flesh

Method n. 925.10 reported by the Association of Official Analytical Chemists procedures was used to evaluate the moisture content [20]. The color of pumpkin pulp powders was measured using the EOPTIS CLM194 colorimeter (Metreo Solutions, Rome, Italy) and the CIELAB scale was used to express the results (L*, a*, b* parameters). The LAB parameters (a* and b*) were used to calculate the LCH parameters (C* and H*, chroma and hue angle, respectively) by applying the EasyRGB colour calculator [21].

### 2.4. Isolation of Carotenoids from Pumpkin Pulp and Evaluation of Total Carotenoid Content

The production of pulp carotenoid extracts, the percentage of extraction (yield %, g/100 g), and the evaluation of their total carotenoid content (TCC) were performed as reported by Pinna et al. (2022) [19]. The TCC was expressed as μg β-carotene equivalents per gram of pumpkin powder (μg β-CE/g).

### 2.5. Cell Culture Conditions

A cytotoxicity assay was carried out on human glioblastoma SH-SY5Y cells. The cells, maintained at 37 °C in a humidified incubator with 5% CO_2_, were cultured in the DMEM supplemented with 10% heat-inactivated FBS, penicillin (100 IU/mL), streptomycin (100 µg/mL), and 1× L-glutamine. Cells were sub-cultured when confluency occurred. All experiments were carried out using passages between 20 and 25.

#### Cell Count and Viability: AO/DAPI Double Staining

SH-SY5Y cells at the density of 1.25 × 10^5^ cells/well were added to a 24-well plate and incubated overnight at 37 °C, in a humidified atmosphere with 5% CO_2_. The dried extract was solubilized in DMSO and then diluted with a culture medium before the treatment. The final content of DMSO never exceeded 0.5% of the culture medium. The treatment was carried out for 24 h using five scalar concentrations of each extract (i.e., 100 μM, 50 μM, 20 μM, 10 μM, 5 μM, 2 μM) starting from the highest soluble concentration of β-carotene (i.e., 100 μM). The two highest concentrations were also tested for 48 h as a further confirmation of the cytotoxicity activity. The molar concentration of extracts was determined using the β-carotene’s molecular weight as a reference, and they correspond to 53.69 μg/mL, 26.84 μg/mL, 10.74 μg/mL, 5.36 μg/mL, 2.68 μg/mL, and 1.07 μg/mL. Briefly, after treatment both the supernatant and cells were collected, centrifuged at 500× *g*, and then suspended in a fresh medium. Thus, aliquots of cell suspensions were stained with AO/DAPI solution and loaded in an NC-Slide A8. The slides were then placed in a NucleoCounter^®^ NC-3000™ (Chemometec, Allerød, Denmark) cytometer interfaced with NucleoView software (http://chemometec.com). Cell viability was calculated as a percentage of the untreated control [22]. At least three independent experiments were conducted for each experimental point. The AO/DAPI staining method for evaluating cytotoxicity was chosen as it does not interfere with colored extracts. AO and DAPI are both nucleic acid-selective fluorescent dyes. AO stains the entire population of cells emitting a green fluorescence (520 nm) after binding the dsDNA. On the contrary, DAPI penetrates only damaged membranes and emits a blue fluorescence (461 nm) after binding the A-T rich region of the DNA [23,24].

### 2.6. In Vitro Antioxidant Activities by Acellular Assays

#### 2.6.1. Free Radical-Scavenging Activity Using ABTS (ABTS Assay)

The ABTS assay, used to determine the free radical-scavenging properties of the pumpkin extracts, was performed as reported by Pollini et al. (2019) [25]. The antioxidant capacity values were reported as μg Trolox equivalents per gram of pumpkin powder (μg TE/g).

#### 2.6.2. Oxygen Radical Absorbance Capacity Assay (ORAC Assay)

The ORAC assay with fluorescein as the fluorescent probe, used to evaluate the antioxidant capacity of pumpkin extracts, was performed as reported by Persichetti et al. (2014) [26]. The automated ORAC assay was carried out on a high-performance plate reader (FLUOstar Optima, BMG LABTECH, Offenburg, Germany) with an excitation wavelength of 485 nm and an emission wavelength of 520 nm. The ORAC values were expressed as μg TE/g.

### 2.7. HPLC-DAD Analysis of Carotenoids

The HPLC equipment, chromatographic conditions, software for data acquisition, and method validation were reported in a previous paper [19]. The carotenoid quantification was performed with calibration curves of: β-carotene, lutein, and zeaxanthin dipalmitate standard solutions. β-carotene was selected to quantify also α-carotene, when detected. Lutein, the most representative compound of non-esterified carotenoids, was selected for their quantification, and the data were expressed as μg lutein equivalents/g (μg LE/g). Zeaxanthin dipalmitate was selected to quantify mono- and di-esterified carotenoids, and the data were expressed as μg zeaxanthin dipalmitate equivalents/g (μg ZDE/g). Zeaxanthin dipalmitate was small-scale isolated from goji berries using HPLC [27].

### 2.8. LC-HRMS Analysis for Carotenoids Structural Confirmation

The LC-HRMS equipment and chromatographic conditions were reported in a previous paper [28]. The identification of the carotenoids was obtained with a comparison between experimental spectra and online MS and MS/MS spectra libraries (Human Metabolome Database or HMDB and MoNA MassBank of North America), and those reported in previous papers [29,30].

### 2.9. Statistical Analysis

The statistical analysis of the cellular assay was carried out using SPSS 20 (SPSS Inc., Chicago, IL, USA). The normal distribution of data was tested using the Shapiro–Wilk test; the groups were then compared by ANOVA, followed by a Dunnett’s post hoc test (a *p*-value < 0.05 was considered significant). All the analytical determinations were performed in triplicate, and the results were reported as the mean ± standard deviation (SD) on a dried weight (DW). Data were processed and edited using Microsoft Excel 2016 software (Microsoft Office, Redmond, WA, USA). Statistical significance among the eight varieties was determined by one-way analysis of variance (ANOVA), followed by Tukey’s honestly significant difference post hoc (a *p*-value < 0.01 was considered significant). The differences between the pumpkin species were measured using a Student’s *t*-test (a *p*-value < 0.05 was considered significant). Chemometric analysis (Principal component analysis -PCA-) and Cluster analysis (Hierarchical Clustering Dendrogram and HeatMap) were obtained using the MetaboAnalyst 6.0 web platform on the autoscaled and normalized dataset [31].

## 3. Results and Discussion

### 3.1. General Characteristics of Pumpkin Cultivars

Cultivated pumpkins are quite similar in terms of growth and development requirements, but the fruit morphology (size, shape, color, and pulp structure) is highly variable [32].

The general characteristics of the pumpkin cultivars tested in this research are presented in Table 1. For all pumpkins, the flesh had a distinct orange color, while the orange skin was shared by the following cultivars: Butternut, Delica vanity, Violina rugosa, Moscata di Provenza, and Hokkaido; in turn, Delica and Lunga di Napoli pumpkins showed a green skin.

Table 2 shows the results of the CIELAB and CIELCH color characteristics of examined pumpkin varieties. This determination was useful to better characterize the pumpkin species/varieties and then to evaluate a possible correlation with analytical data of the obtained extracts.

Based on these measurements, a wide range of values can be observed: L* (26.98–56.87), a* (2.58–14.98), b* (25.19–49.28), C* (27.28–49.52), and H* (66.21–86.96) parameters. All values of a* and b* were on the positive scales, suggesting that the carotenoid extracts were red and yellow on the first quadrant in the LAB/LCH color space. The value of H* was in the first quadrant of the hue angle (0–90°) and located in the range of red hue to yellow hue. No statistically different results (*p* > 0.05) were obtained comparing the color data of the two varieties *C. moschata* vs. *C. maxima* species. The results of the color parameters (L*, a*, b*, C*, H*) were processed to evaluate the degree of correlation (Table S1). As regards *C. maxima*, very good coefficients of correlation (R^2^ ≥ 0.9171) were obtained, while for *C. moschata* the most interesting correlation values were reported for L* vs. a* (R^2^ = 0.9822) and L* vs. H (R^2^ = 0.9919). Other authors have reported a wide variation in the color data [10,33,34]. Kulczyński et al. (2020) showed for the pulp of pumpkin varieties belonging to *C. moschata* and *C. pepo* species the following values: L* (52.00–71.98), a* (−5.44–30.84), and b* (29.24–51.84) parameters [10]. Other studies [33] also found differences in the color range between pumpkins belonging to three different species: *C. maxima* (L* = 36.08, a* = 3.30, b* = 17.60), *C. pepo* (32.67, a* = −0.13, b* = 1.68), and *C. moschata* (L* = 35.58, a* = 0.52, b* = 11.24). Paciulli et al. (2019) reported a deep colorimetric characterization of Delica and Butternut pumpkin species as raw samples and after high-pressure treatments, showing interesting color changes [34]. Also, Norfezah et al. (2011) reported a change in pulp colour parameters after pumpkin flour production by cabinet drying (from 67.94 to 75.84 for L*; from 12.29 to 5.77 for a*; from 42.75 to 37.76 for b*) for a mature Crown pumpkin (*C. maxima*) [35]. C* and H* values ranged respectively from 31.9 to 72.2, and from 77.5 to 99.0 for pumpkin cultivars belonging to the *C. moschata* species [36]. The pumpkin pulp moisture was also determined and values between 81.65 and 96.86% for the *C. moschata* species and between 83.34 and 89.61% for the *C. maxima* species were obtained. Statistically different results were obtained from the comparison between moisture values of the two species (*p* < 0.05), but also considering different varieties belonging to the same species (*p* < 0.01). Other authors have also reported similar values. Norfezah et al. (2011) reported a moisture value of 84.34% for the *C. maxima* species [35], while Karanja et al. (2014) showed a range from 75.08% to 91.16% for 13 groups of pumpkins cultivated in Kenya [37].

### 3.2. Cell Count and Viability: AO/DAPI Double Staining

An optimal extraction procedure, providing efficient extraction and limiting the chemical decomposition and biological activity modification of bioactive compounds, must be followed to evaluate the cytotoxicity of pumpkin carotenoids on neuroblastoma cells [38]. Based on a previous paper, an unconventional extraction technique (UAE), and the hexane:isopropanol (60:40 *v*/*v*) mixture were used to perform the isolation of carotenoids from pumpkin pulp, belonging to two species/eight varieties [19]. The neuroprotective potential of carotenoid extracts has been largely examined. Feng et al. (2016) showed that lycopene exerts neuroprotective effects against apoptosis, oxidative stress and mitochondrial dysfunction [39]. Moreover, the ability to improve the redox status by modulating the Nrf2 transcriptional activity of carotenoids such as fucoxanthin and fucoxanthinol in SH-SY5Y, has been well described [40]. A metabolomic study indicated that treatment with carotenoid extracts can alter the lipidomic composition of SH-SY5Y cells contributing to the neuroprotective effect against toxic agents. Interestingly, docking studies revealed the interaction via hydrogen bonding and van der Waals interactions between carotenoids and Ab peptide from extracellular medium, preventing neurotoxic aggregation and accumulation, and cell death [41]. Huang et al. (2017), found that carotenoids also significantly reduce Ab1-42 secretion in SH-SY5Y acting as potential molecules against Alzheimer’s disease features [42].

**Figure 1:**
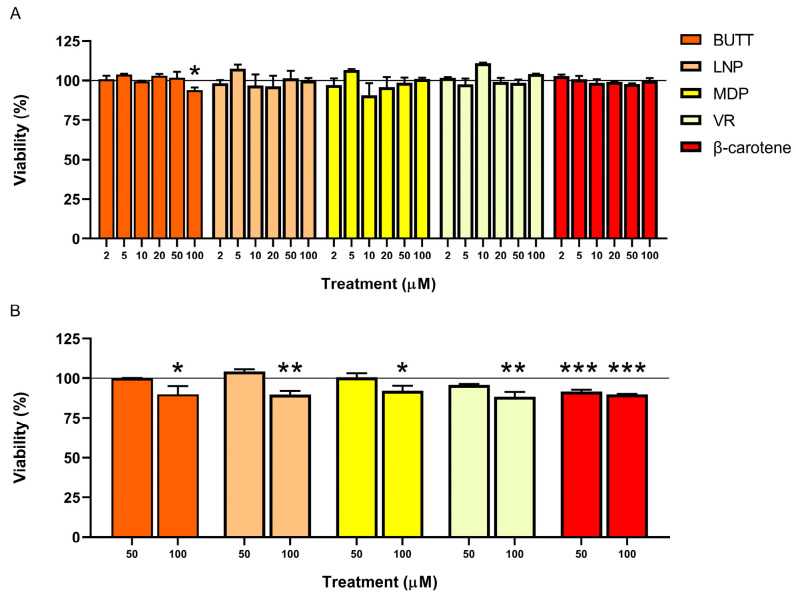
Effects of scalar concentrations of pumpkin (*C. moschata* species) pulp extracts on SH-SY5Y viability. Experimental groups comprised cells treated for 24 (**A**) and 48 h (**B**). The results of each experimental set are expressed as percentage of negative control (taken as unit, 100%), and summarized as the mean ± standard error of the mean of at least three independent experiments. Statistical analysis: one-way ANOVA followed by Dunnett’s post hoc analysis. * (*p* < 0.05), ** (*p* < 0.01), or *** (*p* < 0.0001). BUTT, Butternut; LNP, Lunga di Napoli; MDP, Moscata di Provenza; VR, Violina rugosa.

**Figure 2:**
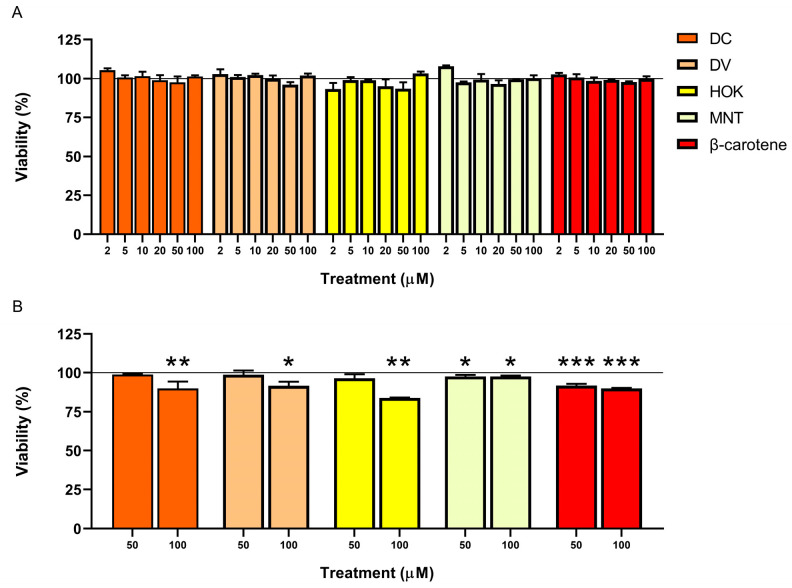
Effects of scalar concentrations of pumpkin (*C. maxima* species) pulp extracts on SH-SY5Y viability. Experimental groups comprised cells treated for 24 (**A**) and 48 h (**B**). The results of each experimental set are expressed as percentage of negative control (taken as unit, 100%), and summarized as the mean ± standard error of the mean of at least three independent experiments. Statistical analysis: one-way ANOVA followed by Dunnett’s post hoc analysis. * (*p* < 0.05), ** (*p* < 0.01), or *** (*p* < 0.0001). DC, Delica; DV, Delica vanity; HP, Hokkaido; MNT, Mantovana.

This study evaluated the viability of SH-SY5Y human neuroblastoma cells after exposure to six increasing concentrations of carotenoid pumpkin extracts. In addition, SH-SY5Y cells were challenged with the same concentration of β-carotene used as a positive control. A total/dead cell double-staining technique was employed using AO and DAPI fluorochromes. Results were expressed as a percentage variation of cell viability concerning the untreated control. After 24 h of treatment (Figure 1A and Figure 2A), only the highest concentration of Butternut (i.e., 100 μM) caused a significant decrease in viability percentage compared to the untreated control (*p* = 0.043). On the contrary, after 48 h of treatment (Figure 1B and Figure 2B), all extracts promoted cell death at a 100 μM concentration (Butternut, *p* = 0.016; Delica, *p* = 0.008; Delica Vanity, *p* = 0.024; Hokkaido, *p* = 0.001; Lunga di Napoli, *p* = 0.001; Mantovana, *p* = 0.035; Moscata di Provenza, *p* = 0.037; Violina rugosa, *p* = 0.001; β-carotene, *p* < 0.0001), whereas both Mantovana and β-carotene showed cytotoxicity at 50 μM (*p* = 0.038 and *p* < 0.0001, respectively).

These findings are in line with a previous paper in which β-carotene at 60 μg/mL (i.e., 112 μM) was reported to induce apoptosis in SH-SY5Y cells through increasing intracellular reactive oxygen species (ROS) production [43]. Indeed, in certain doses, almost all antioxidants can behave as prooxidants, triggering cell death pathways mediated by increased ROS production [12]. The current study indicates that pulp pumpkin extracts exert cytotoxic action against SH-SY5Y neuroblastoma cells, laying an attractive basis for further research. Several investigations will be required to determine the mechanism underlying the extract’s anti-cancer activity, most likely owing to the synergistic effect of several compounds in the phytocomplex. Moreover, in vivo studies are needed to evaluate the effects of carotenoid extracts on living organisms.

### 3.3. TCC and Antioxidant Activity of Pumpkin Pulp

The tested pumpkin extracts were characterized for their carotenoid content and antioxidant properties. Table 3 shows the values of the yield of carotenoid extraction, TCC, and in vitro antioxidant activity of pulp extracts. The percentage of extraction (yield, %), determined by the gravimetric method, was calculated using the equation reported in a previous paper [28]. It is possible to observe a wide range of values even if the same pumpkin species were considered (i.e., from 1.03% of Delica vanity to 4.15% of Delica). In addition, no statistical differences (*p* > 0.05) were found between *C. moschata* and *C. maxima* species, even if the same extraction method and conditions (solvent, temperature, time, solid/liquid ratio) were used. After that, a spectrophotometric characterization was carried out to determine the TCC and antioxidant properties of the extracts. The TCC results of the considered eight varieties change over the range 161.08 μg/g of Butternut to 443.89 μg/g of Violina rugosa, both varieties belonging to the *C. moschata* species, while for *C. maxima* the values ranged from 241.32 to 379.36 μg/g (Delica vanity and Delica, respectively). To make an interesting comparison with the literature, it must be taken into consideration that TCC values, as well as biological properties, could change from pumpkin variety to variety, or in the same variety, they could change based on the extraction method and conditions and many other variables [9,19].

The TCC values reported in this paper are similar to those found by other authors, for example de Carvalho et al. (2012) [44]. They found a range of TCC from 234.21 to 404.98 μg/g in two landrace pumpkins (*C. moschata*) cultivated in Brazil. Higher or lower values have been found compared to other papers. For example, Armesto et al. (2020) reported TCC levels of 34.54–39.53 μg/g for Butternut (*C. moschata*) pumpkin from Spain, extracted by UAE with acetone [11]. Hussain et al. (2022) reported a TCC value of 35.2 mg/100 g for flesh powder from *C. maxima* from Pakistan, extracted with 80% methanol, in an orbital shaker for 120 h at room temperature [45]. Biesiada et al. (2011) reported that the highest content of carotenoids was recorded in a cultivar belonging to *C. maxima* from Poland, Amazonka (18.40 mg/100 g fresh weight), while the lowest (0.57 mg/100 g fresh weight) was for *C. pepo*, *Pyza cultivar* [46]. Azizah et al. (2009) evaluated 22 cultivars of *C. moschata* reporting a TCC ranging from 7.02 to 138.56 μg/g, using ethanol for the extraction by a flask placed in a water bath at 25 °C for 1 h [47].

As regards antioxidant activity results, the ABTS values ranged from 280.91 μg TE/g of Butternut to 1192.11 μg TE/g of Delica, while ORAC values ranged from 1267.86 μg TE/g of Delica vanity to 3996.18 μg TE/g of Delica. Statistically different results were obtained from the comparison between ABTS values of the *C. moschata* and *C. maxima* species, as well as between ORAC values (*p* < 0.05), but also taking into consideration different varieties belonging to the same species (*p* < 0.01). The results of spectrophotometric analyses were processed to evaluate their degree of correlation (Table S2), and a good positive linear relationship between all parameters (R^2^ ≥ 0.5666) was found.

For a literature comparison, it is worth noting that some papers reported ABTS and ORAC values of extracts obtained with polar solvents (methanol, water), where the antioxidant activity was probably linked to polyphenols, but not to carotenoids. As an example, Kulczyński et al. (2020) reported for Butternut lower ABTS values both taking into consideration aqueous-methanol extract (126.92 mg TE/100 g) and aqueous extract (138.36 mg TE/100 g), and found the lowest ABTS values for Futsu and Table Queen pumpkin cultivars [10]. Regarding ORAC values, the same authors found the lowest and the highest values for cultivars belonging to the C. pepo (i.e., Table Queen 28.12 μmol TE/g; Delicata 108.3 μmol TE/g). Only a few papers reported the values of antioxidant assays on carotenoid extracts. As an example, Pinna et al. (2022) reported values of 958.88 and 2832.76 μg TE/g for ABTS and ORAC, respectively, for extracts of *C. moschata* obtained with hexane:isopropanol (60:40 *v*/*v*) [19].

### 3.4. Carotenoid Composition of Pulp and Multivariate Statistical Analysis

Having established that the pumpkin extract, characterized by low polarity compounds, showed interesting biological activity, the next fundamental step was the in-depth characterization of the main fraction, i.e., carotenoid compounds.

A qualitative and quantitative analysis of carotenoids by an HPLC-DAD procedure was performed, using a method validated in previous works [19,28]. A UHPLC-MS/MS technique was also carried out for the structural confirmation of the analytes.

The quantitation of identified carotenoids was based on calibration curves obtained using lutein and zeaxanthin dipalmitate standard solutions (Table S3). The calibration curve of β-carotene and the relative values of linearity and accuracy were reported in a previous paper [19]. The β-carotene standard was used for the quantification of non-esterified cyclic carotenes (β-carotene and α-carotene); other non-esterified carotenoids (i.e., violaxanthin, antheraxanthin, neoxanthin, lutein, zeaxanthin) were quantified using the regression equation of lutein (expressed as μg LE/g DW), while esterified (mono- and di-) carotenoids (which included violaxanthin and antheraxanthin myristate, lutein and antheraxanthin palmitate, as well as violaxanthin di-myristate, antheraxanthin di-laurate and others) were quantified using the regression equation of zeaxanthin dipalmitate (expressed as μg ZDE/g DW). The regression model showed good linearity for lutein and zeaxanthin dipalmitate (R^2^ > 0.996 and 0.994, respectively). Furthermore, values of accuracy, precision, limit of detection, and quantification (LOD and LOQ, respectively) were measured to validate the RP-HPLC method (Table S4). This internal validation, performed at a basic level to demonstrate the suitability of the in-house developed method, provided useful results, thus ensuring that the process is satisfactory and consistent within the scope of the present study. Table S5 shows the UV-Vis and mass spectral data used for the carotenoid identification.

**Figure 3:**
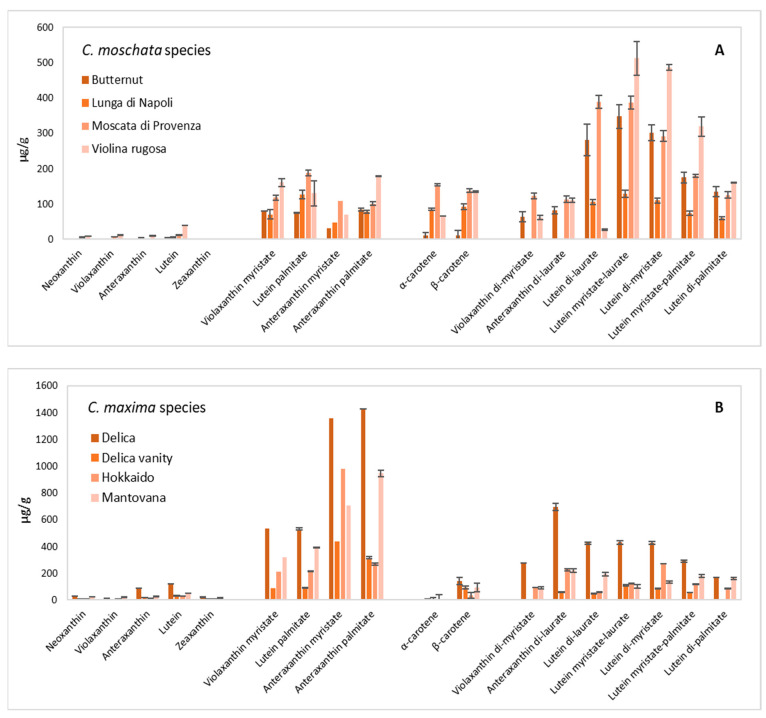
Content of pulp carotenoids (μg/g) of the pumpkin varieties belonging to *C. moschata* (**A**) and *C. maxima* (**B**) species.

**Figure 4:**
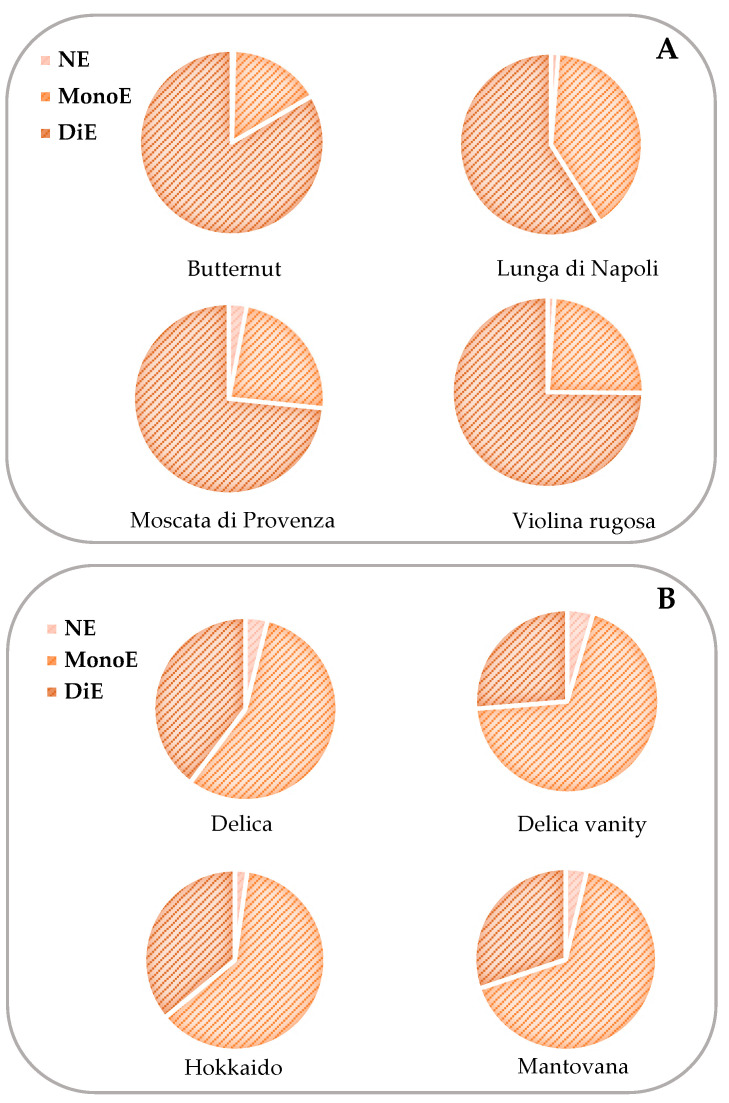
Content of pulp carotenoids (μg/g), grouped as non-esterified (NE), monoesterified (MonoE) and diesterified (DiE) of the pumpkin varieties belonging to *C. moschata* (**A**) and *C. maxima* (**B**) species.

Figure 3A,B shows the data of carotenoid composition, while Figure 4A,B shows the content of grouped carotenoids. The most abundant xanthophylls in pumpkin pulp were monoesterified for *C. maxima* and diesterified for *C. moschata*. A study of correlation between spectrophotometric and chromatographic data was carried out. It can be noted that *C. maxima* data always showed high values of R^2^ which were between 0.5411 and 0.9907 (Table S2). The cultivars with the highest β-carotene content were Delica, Violina rugosa, and Moscata di Provenza (134.52–140.38 μg/g), while α-carotene was only detected in pumpkin belonging to the *C. moschata* species (10.29–153.86 μg/g). Generally, it should be noted that carotenoid content varied in a wide range [8,38,39,41], also considering the dependence of this data on cultivar, variety, harvesting time, storage, and processing. Hussain et al. (2022) reported a β-carotene content of 6.18 mg/100 g for flesh pumpkin (*C. maxima*) [45]. De Carvalho et al. (2012) found that all E-β-carotene was the most abundant isomer, varying from 141.95 to 244.22 μg/g, compared to α-carotene (67.06–72.99 μg/g), while 9- and 13-Z-β-carotene isomers were found in low concentrations [44]. Kreck et al. (2006) reported that pumpkin varieties belonging to *C. maxima* differ significantly in terms of carotenoids; in fact, the concentration of β-carotene ranged from 17 mg/kg to 263 mg/kg [48]. Murkovic et al. (2002) reported a content of α-carotene from 0.03 mg/100 g for *C. pepo* (Carnevale di Venezia variety) up to 7.5 mg/100 g for *C. maxima* (Flat White Boer variety) and a content of β-carotene from 0.06 to 7.4 mg/100 g [49]. Dhenge et al. (2022) reported the content of carotenoids after high-pressure processing (α-carotene 29.2–78.3 μg/g; β-carotene 10.5–20.4 μg/g) [50]. Recently, Grassino et al. (2023) reviewed the carotenoid contents and profiles of pumpkin products and by-products. They reported β-carotene values of 5.70 and 17.04 μg/g for the pulp of *C. moschata* and *C. maxima*, respectively. The content of α-carotene was reported only for pumpkin seed oil, puree, juice, and extrudates [51].

In this research, in addition to cyclic carotenes (α- and β-carotene), other non-esterified carotenoids were detected, among which were epoxycarotenoids (neoxanthin, violaxanthin, and antheraxanthin) and hydroxycarotenoids (zeaxanthin and lutein). The Delica and Mantovana varieties, belonging to the *C. maxima* species, showed the highest contents (266.195 and 135.23 μg LE/g, respectively). The Butternut and Lunga di Napoli varieties of the *C. moschata* species showed the lowest content (6.55 and 10.22 μg LE/g, respectively) of this class of non-esterified compounds. However, there was a notable difference within the single compound content (neoxanthin, violaxanthin, antheraxanthin, lutein, and zeaxanthin). As regards the lutein content, in this work the values ranged from 5.24 μg LE/g of Butternut up to 119.26 μg LE/g of Delica. Generally, it can be observed that this last variety was the one richest in carotenoid content; in fact, it showed the highest content of β-carotene, as well as non-esterified and esterified carotenoids. Kulczyński and Gramza-Michałowska (2019) reported a lutein content ranging from 87.20 μg/g for the Porcelain Doll to 388.79 for the Melonowa Żółta variety, with a value of 130.23 μg/g for Hokkaido [8]. A lutein content from 0.6 to 17.3 μg/g fresh weight was reported by Itle and Kabelka (2009) [36].

Among hydroxycarotenoids, zeaxanthin was quantified by some authors with a wide range of contents [8,29,45]. Kurz et al. (2008) reported values of 0.57 μg/g for the Halloween pumpkin to 22.45 μg/g for the Hokkaido variety [29], while Kulczyński and Gramza-Michałowska (2019) reported from 19.57 μg/g for the Buttercup to 192.53 μg/g for the Melonowa Żółta variety [8]. In the research of Murkovic et al. (2002), lutein and zeaxanthin were not separated by a routine HPLC method, so the authors reported that the content of lutein (+ zeaxanthin) changed from 0.8 to 17 mg/100 g for the *C. maxima* species, and from 0.08 to 1.1 mg/100 g for the *C. moschata* species [49]. In this deep characterization of the carotenoid fraction of pumpkin, it was found that many carotenoids were linked to saturated and long-chain fatty acids, i.e., lauric (C12:0), myristic (C14:0), and palmitic (C16:0) acids.

Among esterified carotenoids, monoesterified carotenoids (i.e., violaxanthin and antheraxanthin myristate, lutein and antheraxanthin palmitate) and diesterified carotenoids (i.e., violaxanthin or lutein di-myristate, antheraxanthin di-laurate, lutein di-palmitate, lutein myristate-laurate, and zeaxanthin myristate-palmitate) were identified and quantified. The presence of fatty esters of xanthophylls, especially of violaxanthin (monomyristate) and lutein (monomyristate and monopalmitate), was published for the first time in 1988 by the group of Khachik [52], even if they did not quantify these compounds since no reference materials were available. Recently, Ouyang et al. (2022) studied the stability of carotenoids and carotenoid esters in pumpkin (*C. maxima*) slices during hot air drying. They identified and quantified six esterified carotenoids, besides free carotenoids. They reported essentially esterified forms of lutein with contents of 20.3 μg/g DW for lutein-stearate-palmitate up to 93.1 μg/g DW for lutein-palmitate-laurate, in addition to 37.8 μg/g DW of violaxanthin-dipalmitate [53].

The esterification of carotenoids was linked to higher stability and potentially higher or equivalent bioavailability compared to free carotenoids; in fact, it has been reported that xanthophyll esters probably need to be hydrolyzed during digestion before absorption and that the in vivo absorption of carotenoids is improved when esterified rather than non-esterified [54,55]. In the conclusion to this section, it must be emphasized that the presence of a high content of carotenoids in pumpkin pulp plays an essential role in maintaining a healthy status, due to the broad spectrum of health-promoting effects of these bioactives, having neuroprotective, ophthalmological, antimicrobial, cardioprotective, antiplasmodial, and skin effects. For example, for age-related eye disease (macular degeneration and cataract), it is sufficient to mention β-carotene, lutein, and zeaxanthin. The first is a precursor of 11-cis retinal, a chromophore of rhodopsin present in rod cells, receptors that allow vision in low-light conditions. The others are the main antioxidant compounds of the retina. They absorb UV radiation and scavenge free radicals, including ROS. For this reason, age-related eye diseases can be prevented and treated with carotenoid supplementation [56,57].

In this paper, to evaluate the possibility that carotenoids could represent valid biomarkers for pumpkin species differentiation (*C. moschata* vs. *C. maxima*), principal component analysis (PCA) was applied and the dataset was represented by the carotenoid content obtained by HPLC-DAD analysis.

**Figure 5:**
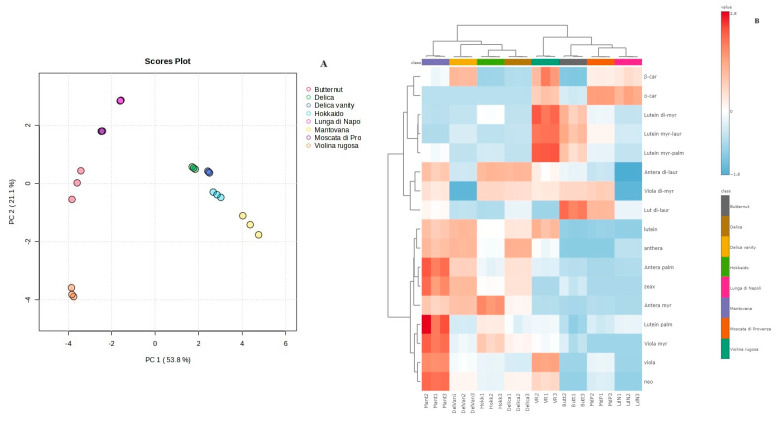
Chemometric analysis and hierarchical clustering of carotenoids of eight pumpkin varieties. Principal Component Analysis 2D scores plot (**A**) and HeatMap (**B**).

Based on 2D scores plot (Figure 5A) and a biplot (Figure S2), the samples were grouped considering the varieties and the species. In particular, the samples belonging to the *C. maxima* species (on the right) were separated from those of *C. moschata* (on the left). To explain the variance, the first two principal components (PC1 and PC2) were extracted, accounting for 74.9% of the variance (PC1: 53.8%; PC2: 21.1%). Figure S3 shows the PCA overview with the pairwise score plot for the top 5 PC; Figure S4 shows the PCA scree plot, indicating the variance explained by the individual PC and the accumulated variance. The HeatMap (Figure 5B) shows that 17 carotenoids were identified as interesting biomarkers. In particular, Violina rugosa showed lutein di-myristate, lutein myristate-laurate, and lutein myristate-palmitate as overexpressed carotenoids; lutein palmitate, violaxanthin-myristate, violaxanthin, and neoxanthin were overexpressed in the Mantovana variety. Antheraxanthin myristate and lutein di-laurate were mainly represented in Hokkaido and Butternut, respectively. Moreover, the hierarchical clustering dendrogram (Figure S5), the branching diagram representing the relationships of similarity between groups, highlighted the clear separation of pumpkin varieties belonging to the two different species (*C. moschata* vs. *C. maxima*).

## 4. Conclusions

In this work, carotenoid extracts from pulp pumpkin of different varieties were assayed for their cytotoxic effect on human neuroblastoma SH-SY5Y and then deeply characterized for their antioxidant properties and chemical profile. Interestingly, as far as the biological study is concerned, at all the tested concentrations after 48 h the pulp pumpkin extracts exert cytotoxic action against SH-SY5Y neuroblastoma cells, laying an attractive basis for further research. The data collected in this research could be useful for a deeper investigation of still scarcely explored biochemical processes modulated by carotenoids regarding cytotoxic effects on human neuroblastoma. Moreover, the results obtained in this study valorized pumpkin as an excellent dietary source of antioxidants. Besides the spectrophotometric measurement of TCC and antioxidant activity, the profiling of carotenoids was deepened by a newly developed HPLC-MS method revealing at least 18 carotenoid compounds. The results of the quantitative analysis showed a high content of β-carotene for Delica and Violina rugosa pumpkins, differently from α-carotene, a typical carotenoid of pumpkins belonging to the *C. moschata* species. Among *C. maxima* species, the Delica variety was the most abundant in carotenoids, while among *C. moschata* species the Violina rugosa variety was. In conclusion, the edible part of the pumpkin is a significant natural source of bioactive substances, showing an intriguing potential against neuroblastoma, an effect that must be confirmed by in vivo studies.

## Figures and Tables

**Table 1:** Main features of pumpkin samples.

Fruit Appearance	** 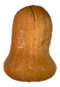 **	** 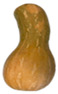 **	** 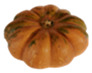 **	** 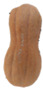 **
*C. moschata* species	Butternut	Lunga di Napoli	Moscata di Provenza	Violina rugosa
Skin color	Orange	Green	Orange with green spot	Orange
Fruit shape	Pear-like shape	Pear-like shape	Round shape	Pear-like shape
Fruit weight, kg	3.318	2.537	5.433	3.120
Flesh color	Orange	Orange	Orange	Orange
Usage	For food use	For food use	For food use	For food use
Rind	Smooth and regular	Smooth and regular	Smooth and regular	Smooth and regular
Harvest time	October	October	October	October
Fruit appearance	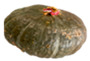	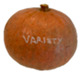	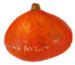	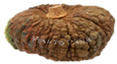
*C. maxima* species	Delica	Delica vanity	Hokkaido	Mantovana
Skin color	Green	Orange	Orange/Red	Brown with green spot
Fruit shape	Round shape	Round shape	Round shape	Round shape
Fruit weight, kg	1.350	1.011	0.539	4.490
Flesh color	Orange	Orange	Orange	Orange
Usage	For food use	For food use	For food use	For food use
Rind	Rough and irregular	Rough and irregular	Smooth and regular	Rough and irregular
Harvest time	October	October	October	October

**Table 2:** Color characteristics (L*, a*, b*, C*, H*) of pumpkin powder.

Cultivar	L*	a*	b*	C*	H*
*C. moschata* species					
Butternut	51.10 ± 0.89 ^a,d^	7.28 ± 0.61 ^a,d^	44.73 ± 0.58 ^a,e^	45.32 ± 0.71 ^a,e^	80.76 ± 0.78 ^a^
Lunga di Napoli	55.58 ± 0.93 ^a,e^	3.97 ± 0.12 ^b^	40.15 ± 1.09 ^a^	40.35 ± 0.87 ^b^	84.35 ± 0.20 ^a,c,d^
Moscata di Provenza	55.17 ± 1.20 ^a,e^	4.87 ± 0.93 ^a,b^	40.98 ± 0.29 ^a^	41.27 ± 0.75 ^a,b^	83.22 ± 1.19 ^a,c,d^
Violina rugosa	34.52 ± 0.85 ^b^	14.98 ± 0.31 ^c^	33.98 ± 0.41 ^b^	37.13 ± 0.33 ^b^	66.21 ± 1.15 ^b^
*C. maxima* species					
Delica	52.71 ± 1.08 ^a,e^	4.87 ± 0.21 ^a,b^	49.28 ± 0.89 ^c,e^	49.52 ± 0.97 ^c,e^	84.36 ± 1.51 ^c,d^
Delica vanity	26.98 ± 1.08 ^c^	10.47 ± 0.42 ^d^	25.19 ± 1.13 ^d^	27.28 ± 1.22 ^d^	67.43 ± 1.94 ^b^
Hokkaido	45.98 ± 1.95 ^d^	5.18 ± 0.18 ^a,b^	41.58 ± 2.43 ^a^	41.90 ± 1.97 ^a,b^	82.90 ± 1.08 ^c^
Mantovana	56.87 ± 2.47 ^e^	2.58 ± 0.05 ^b^	48.62 ± 1.77 ^e^	48.69 ± 1.15 ^e^	86.96 ± 2.03 ^d^

Data are reported as mean value ± SD of three independent measurements (n = 3) and are expressed on dry weight. L*, color lightness; a*, color in the range from green (negative) to red (positive); b*, color from blue (negative) to yellow (positive); C*, Chroma; H*, Hue angle. Different letters in each column indicate significant differences with *p* < 0.01.

**Table 3:** Values of extraction yield, TCC and in vitro antioxidant activity of pulp extracts.

Cultivar	Yield (%)	TCC (μg β-CE/g)	ABTS (μg TE/g)	ORAC (μg TE/g)
*C. moschata* species				
Butternut	1.63 ± 0.20 ^a,b^	161.08 ± 7.80 ^a^	280.91 ± 27.45 ^a^	1352.34 ± 10.34 ^a^
Lunga di Napoli	1.29 ± 0.06 ^a,d^	303.27 ± 2.08 ^b^	343.13 ± 18.82 ^a,b^	1802.54 ± 76.54 ^a,b^
Moscata di Provenza	2.00 ± 0.25 ^b,e^	365.73 ± 9.49 ^c^	417.62 ± 53.94 ^b,c,e^	1500.30 ± 53.04 ^a,d^
Violina rugosa	1.42 ± 0.15 ^a,d^	443.89 ± 7.58 ^d^	525.39 ± 32.24 ^c,f^	2560.11 ± 324.24 ^b^
*C. maxima* species				
Delica	4.15 ± 0.00 ^c^	379.36 ± 44.08 ^c^	1192.11 ± 48.44 ^d^	3996.18 ± 72.58 ^c^
Delica vanity	1.03 ± 0.01 ^d^	241.32 ± 21.55 ^e^	313.23 ± 16.22 ^a,e^	1267.86 ± 166.22 ^a^
Hokkaido	1.58 ± 0.06 ^a,b^	310.77 ± 23.82 ^b^	548.41 ± 68.45 ^f^	2341.24 ± 68.45 ^b,d^
Mantovana	2.20 ± 0.28 ^e^	247.05 ± 13.62 ^e^	500.90 ± 11.69 ^c,f^	2615.96 ± 52.10 ^b^

Mean value ± SD of three independent measurements (n = 3) are expressed on dry weight. TCC, total carotenoid content; ABTS, 2,20-azino-bis(3-ethylbenzothiazoline-6-sulfonic acid) diammonium salt; ORAC, oxygen radical absorbance capacity; TE, Trolox equivalents; β-CE, β-carotene equivalents. Different letters in each column indicate significant differences with *p* < 0.01.

## Data Availability

The original contributions presented in the study are included in the article/Supplementary Material, further inquiries can be directed to the corresponding author.

## Supplementary Materials

The following supporting information can be downloaded at: https://www.mdpi.com/article/10.3390/nu16173043/s1, Figure S1: A typical HPLC-DAD chromatographic profile of pumpkin pulp. A, Hokkaido variety (*C. maxima* species); B, Moscata di Provenza variety (*C. moschata* species). 1, neoxanthin; 2, violaxanthin; 3, antheraxanthin; 4, lutein; 5, zeaxanthin; 6, violaxanthin myristate; 7, lutein palmitate; 8, antheraxanthin myristate; 9, antheraxanthin palmitate; 10, α-carotene; 11, β-carotene; 12 violaxanthin di-myristate; 13, antheraxanthin di-laurate; 14, lutein di-laurate; 15, lutein laurate myristate; 16, lutein di-myristate; 17, lutein palmitate myristate; 18, lutein di-palmitate; Figure S2: PCA overview. Display pairwise score plot for top 5 PCs; Figure S3: PCA scree plot. The green line on top shows the accumulated variance explained; the blue line underneath shows the variance explained by individual PC. Figure S4: Biplot for principal components (PC1 and PC2); Figure S5: Hierarchical Clustering Dendrogram. Distance Measure: Euclidean; Clustering Algorithm: Ward; Table S1: Correlation between color parameters (L*, a*, b*, C*, H*) of two different species (*C. moschata* and *C. maxima*); Table S2: Correlation between spectrophotometric parameters (TCC, ABTS, ORAC) of two different species (*C. moschata* and *C. maxima*); Table S3: Regression equation, R^2^, linearity range, LOD, and LOQ of lutein and zeaxanthin dipalmitate, analyzed by HPLC-DAD; Table S4: Method validation for lutein and zeaxanthin dipalmitate: evaluation of precision (RSD %) and accuracy (recovery %) in the short- and long-term period (intra-day and inter-day precision and accuracy values); Table S5: HPLC retention times, ultraviolet (UV)/visible light (Vis) spectra, and MS spectral data of carotenoids from pumpkin pulp.
