# Supplementary material for: Carotenoids from Different Pumpkin Varieties Exert a Cytotoxic Effect on Human Neuroblastoma SH-SY5Y Cells
Source: Nutrients. 2024 Sep 9;16(17):3043. doi: 10.3390/nu16173043 (PMC11397300; doi:10.3390/nu16173043)
Supplement: Supplementary file 1 [file nutrients-16-03043-s001.zip › nutrients-3156221-supplementary.pdf]

# Carotenoids from Different Pumpkin Varieties Exert a Cytotoxic Effect on Human Neuroblastoma SH-SY5Y Cells

**Nicola Pinna [1], Federica Ianni [1], Carmela Conte [1], Michela Codini [1], Raffaella di Vito [1], Stefania Urbani [2], Roberto Selvaggini [2], Lina Cossignani [1,*] and Francesca Blasi [1]**

[1] Department of Pharmaceutical Sciences, University of Perugia, 06126 Perugia, Italy; nicola.pinna@dottorandi.unipg.it (N.P.); federica.ianni@unipg.it (F.I.); carmela.conte@unipg.it (C.C.); michela.codini@unipg.it (M.C.); raffaella.divito@outlook.it (R.d.V.); francesca.blasi@unipg.it (F.B.)

[2] Department of Agricultural, Food and Environmental Sciences, University of Perugia, 06126 Perugia, Italy; stefania.urbani99@gmail.com (S.U.); roberto.selvaggini@unipg.it (R.S.)

* Correspondence: lina.cossignani@unipg.it; Tel.: +39-075-585-7959

**Table S1.** Correlation between color parameters (L, a, b, C, H) of two different species (*C. moschata* and *C. maxima*).

| | | L* | a* | b* | C* | H* |
|---|---|---|---|---|---|---|
| **L*** | *C. moschata* | - | 0.9822 | 0.5958 | 0.3544 | 0.9919 |
| | *C. maxima* | - | 0.9171 | 0.9561 | 0.9505 | 0.9573 |
| **a*** | *C. moschata* | 0.9822 | - | 0.4948 | 0.2597 | 0.9884 |
| | *C. maxima* | 0.9171 | - | 0.8213 | 0.8056 | 0.9457 |
| **b*** | *C. moschata* | 0.5958 | 0.4948 | - | 0.9403 | |
| | *C. maxima* | 0.9561 | 0.8213 | - | 0.9993 | |
| **C*** | *C. moschata* | 0.3544 | 0.2597 | 0.9403 | - | 0.3572 |
| | *C. maxima* | 0.9505 | 0.8056 | 0.9993 | - | 0.9147 |
| **H*** | *C. moschata* | 0.9919 | 0.9884 | 0.6003 | 0.3572 | 0.6003 |
| | *C. maxima* | 0.9573 | 0.9457 | 0.9286 | 0.9147 | 0.9286 |

**Table S2.** Correlation between spectrophotometric parameters (TCC, ABTS, ORAC) of two different species (*C. moschata* and *C. maxima*).

| | | TCC | ABTS | ORAC |
|---|---|---|---|---|
| **TCC** | *C. moschata* | - | 0.8618 | 0.5666 |
| | *C. maxima* | - | 0.7601 | 0.6122 |
| **ABTS** | *C. moschata* | 0.8618 | - | 0.5980 |
| | *C. maxima* | 0.7601 | - | 0.8513 |
| **ORAC** | *C. moschata* | 0.5666 | 0.5980 | - |
| | *C. maxima* | 0.6122 | 0.8513 | - |

TCC, total carotenoid content measured by spectrophotometric assay; ABTS, 2,2'-azino-bis(3-ethylbenzothiazoline-6-sulfonic acid) diammonium salt; ORAC, Oxygen Radical Absorbance Capacity

**Table S3.** Regression equation, $R^2$, linearity range, LOD, and LOQ of lutein and zeaxanthin dipalmitate, analyzed by HPLC-DAD.

| Standard | Regression Equation | $R^2$ | Linearity range (µg/mL) | LOD (ng/mL) | LOQ (ng/mL) |
|---|---|---|---|---|---|
| Lutein | y = 182.59 (±5.69)x - 36.15 (±20.72) | 0.996 | 0.24 - 5.90 | 0.37 | 1.13 |
| Zeaxanthin dipalmitate | y = 11.03 (±0.44)x -27.66 (±2.34) | 0.994 | 0.95 – 95.0 | 0.70 | 2.12 |

Limit of detection (LOD); limit of quantification (LOQ).

**Table S4.** Method validation for lutein and zeaxanthin dipalmitate: evaluation of precision (RSD %) and accuracy (recovery %) in the short- and log-term period (intra-day and inter-day precision and accuracy values).

| Theoretical conc. (µg/mL) | Intra-day mean conc. (µg/mL) | Intra-day Precision (RSD %) | Intra-day Accuracy (Recovery %) | Inter-day mean conc. (µg/mL) | Inter-day Precision (RSD %) | Inter-day Accuracy (Recovery %) |
|---|---|---|---|---|---|---|
| Lutein | 4.56 | 1.55 | 96.57 | | | |
| 4.72 | 4.75 | 1.70 | 100.68 | 4.71 | 3.18 | 99.86 |
| | 4.83 | 2.93 | 102.32 | | | |
| Zeaxanthin dipalmitate | 72.17 | 2.18 | 100.84 | | | |
| | 72.38 | 3.46 | 101.59 | 71.31 | 2.85 | 100.09 |
| 71.25 | 69.73 | 1.65 | 97.86 | | | |

Intra-day and Inter-day evaluation: analysis of 3 replicates of the selected external standard within one day and for three consecutive days.

**Table S5.** HPLC retention times, ultraviolet (UV)/visible light (Vis) spectra, and MS spectral data of carotenoids from pumpkin pulp.

| Carotenoids | Molecular formula | Rt (min) | λmax (nm) | $[M+H]^+$ (*m/z*) | Fragments (*m/z*) |
|---|---|---|---|---|---|
| neoxanthin | $C_{40}H_{56}O_4$ | 5.05 | 416, 442, 470 | 601 | 583, 565, 221 |
| violaxanthin | $C_{40}H_{56}O_4$ | 5.83 | 420, 444, 472 | 601 | 583, 565, 491 |
| antheraxanthin | $C_{40}H_{56}O_3$ | 8.96 | 445, 473 | 585 | 567, 549, 493 |
| lutein | $C_{40}H_{56}O_2$ | 9.61 | 422sh, 445, 473 | 569 | 551, 553, 463 |
| zeaxanthin | $C_{40}H_{56}O_2$ | 10.53 | 426sh, 450, 477 | 569 | 551, 553 |
| α-carotene | $C_{40}H_{56}$ | 35.07 | 422sh, 445, 473 | 537 | 537, 444, 177 |
| β-carotene | $C_{40}H_{56}$ | 37.80 | 426sh, 450, 477 | 537 | 444, 177 |
| Violaxanthin myristate | $C_{55}H_{84}O_3$ | 27.46 | 416,442,470 | 812 | 794, 776, 565 |
| Lutein palmitate | $C_{56}H_{86}O_3$ | 30.63 | 420, 444, 472 | 809 | 551, 533, 495 |
| Antheraxanthin myristate | $C_{55}H_{84}O_3$ | 33.42 | 420, 446, 472 | 797 | 779, 705 |
| Antheraxanthin palmitate | $C_{56}H_{86}O_5$ | 36.17 | 420, 446, 471 | 825 | 807, 733 |
| Violaxanthin di-myristate | $C_{70}H_{112}O_6$ | 40.13 | 413, 435, 464 | 1022 | 1004, 793, 775 |
| Antheraxanthin di-laurate | $C_{70}H_{112}O_6$ | 42.01 | 418, 442, 470 | 949 | 931, 857, 533 |
| Lutein di-laurate | $C_{70}H_{112}O_4$ | 45.61 | 420, 445, 474 | 942 | 734, 533 |
| Lutein myristate-laurate | $C_{68}H_{108}O_4$ | 47.17 | 421, 443, 474 | 960 | 761, 733, 533 |
| Lutein di-myristate | $C_{71}H_{114}O_4$ | 48.74 | 422, 446, 474 | 990 | 761, 669, 533 |
| Lutein myristate-palmitate | $C_{70}H_{112}O_4$ | 50.32 | 421, 443, 467 | 1018 | 790, 762, 533 |
| Lutein di-palmitate | $C_{72}H_{116}O_4$ | 51.79 | 428, 448, 476 | 1046 | 790, 533 |

:

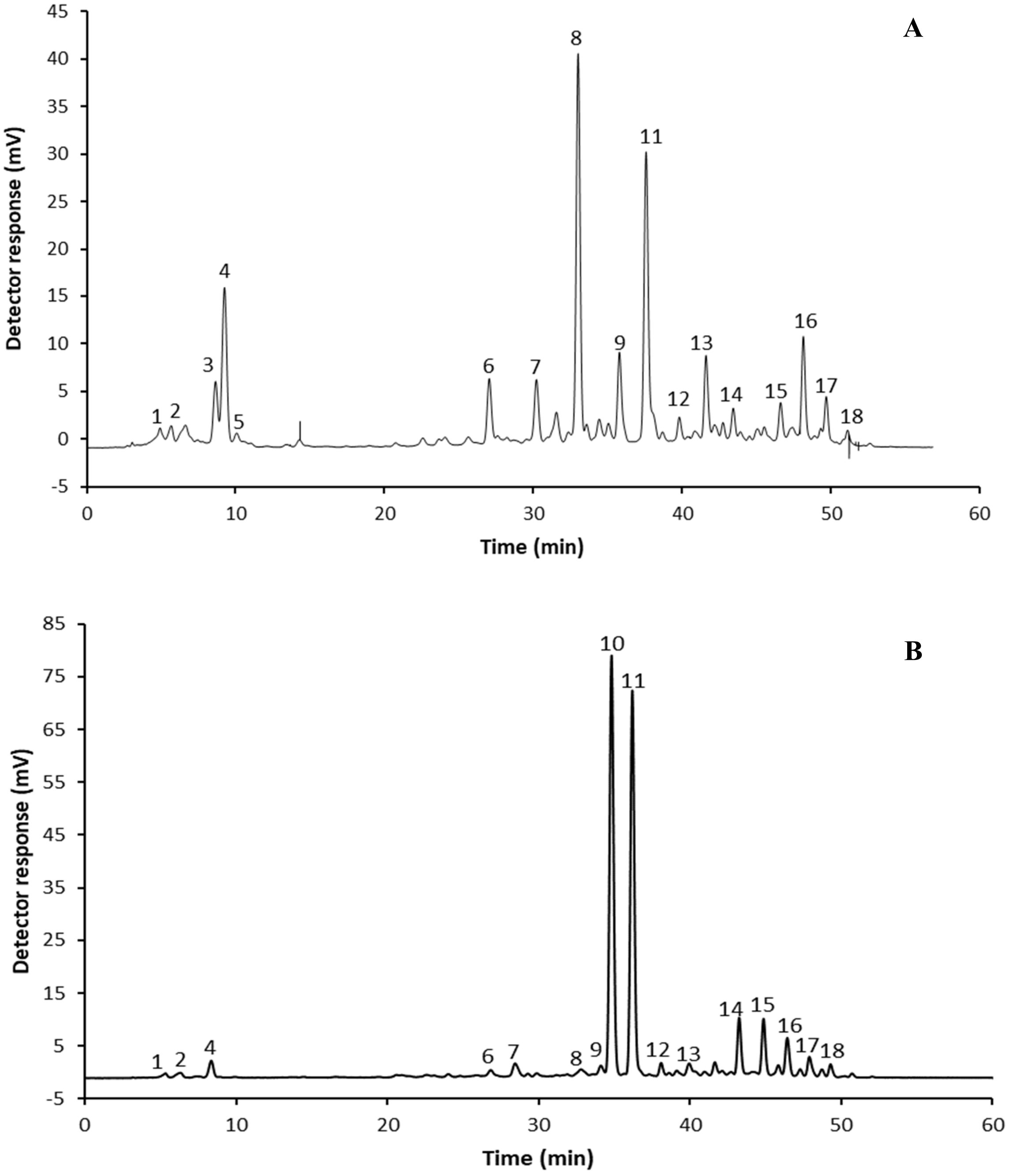


**Figure S1:** A typical HPLC-DAD chromatographic profile of pumpkin pulp. A, Hokkaido variety (*C. maxima* species); B, Moscata di Provenza variety (*C. moschata* species). 1, neoxanthin; 2, violaxanthin; 3, antheraxanthin; 4, lutein; 5, zeaxanthin; 6, violaxanthin myristate; 7, lutein palmitate; 8, antheraxanthin myristate; 9, antheraxanthin palmitate; 10, α-carotene; 11, β-carotene; 12 violaxanthin di-myristate; 13, antheraxanthin di-laurate; 14, lutein di-laurate; 15, lutein laurate myristate; 16, lutein di-myristate; 17, lutein palmitate myristate; 18, lutein di-palmitate.

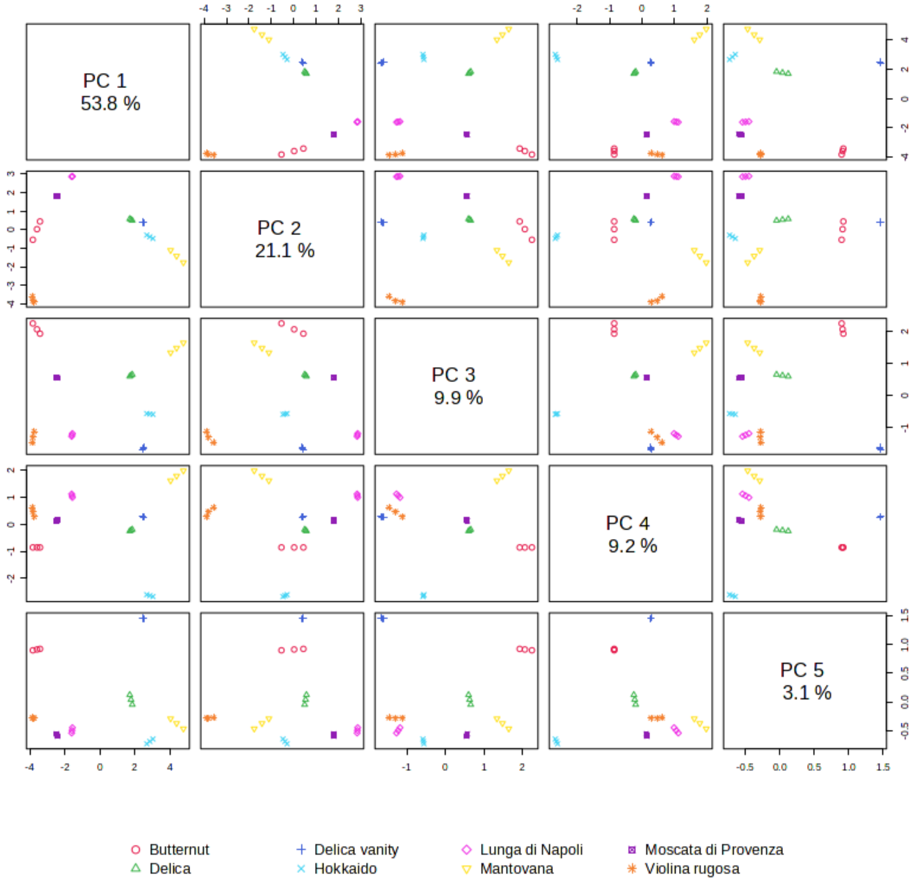


**Figure S2:** PCA overview. Display pairwise score plot for top 5 PCs.

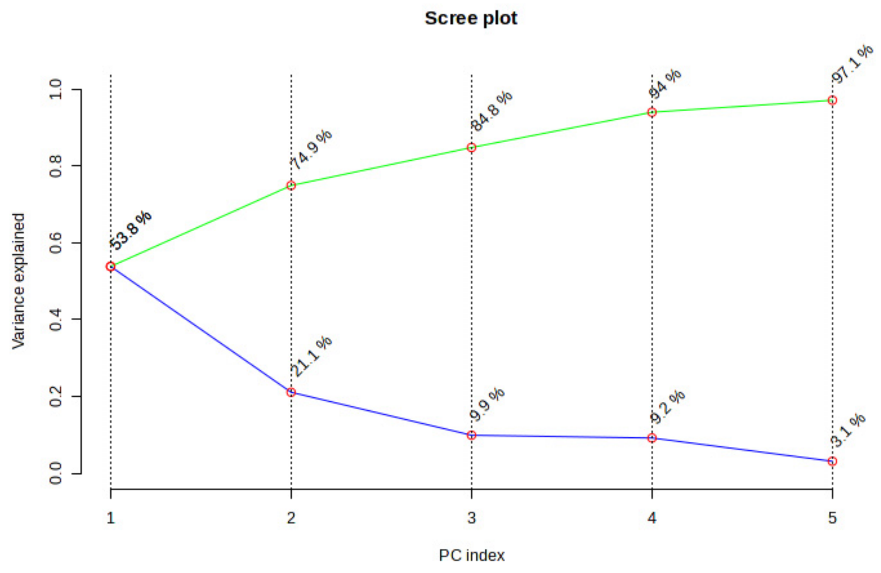


**Figure S3:** PCA scree plot. The green line on top shows the accumulated variance explained; the blue line underneath shows the variance explained by individual PC.

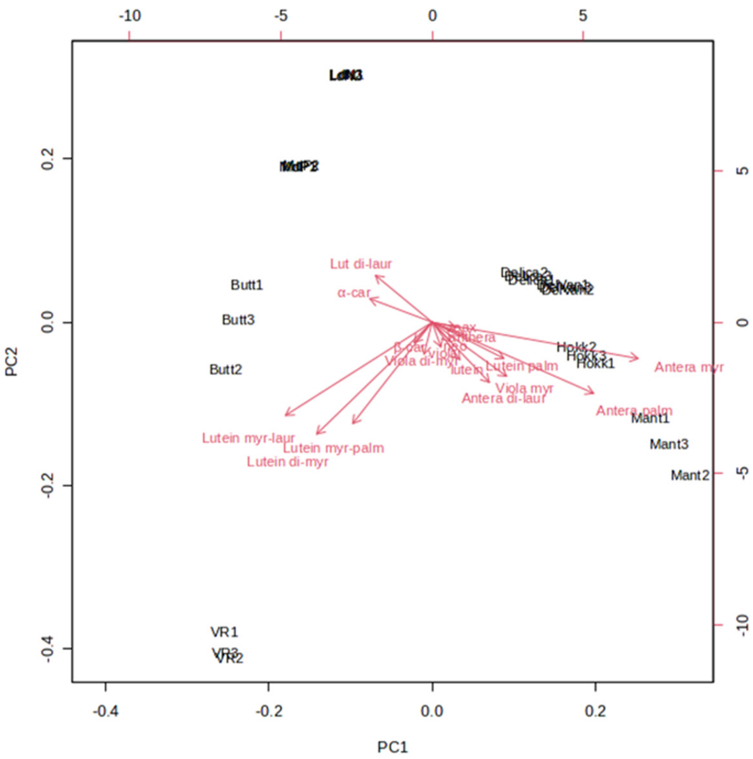


**Figure S4:** Biplot for principal components (PC1 and PC2).

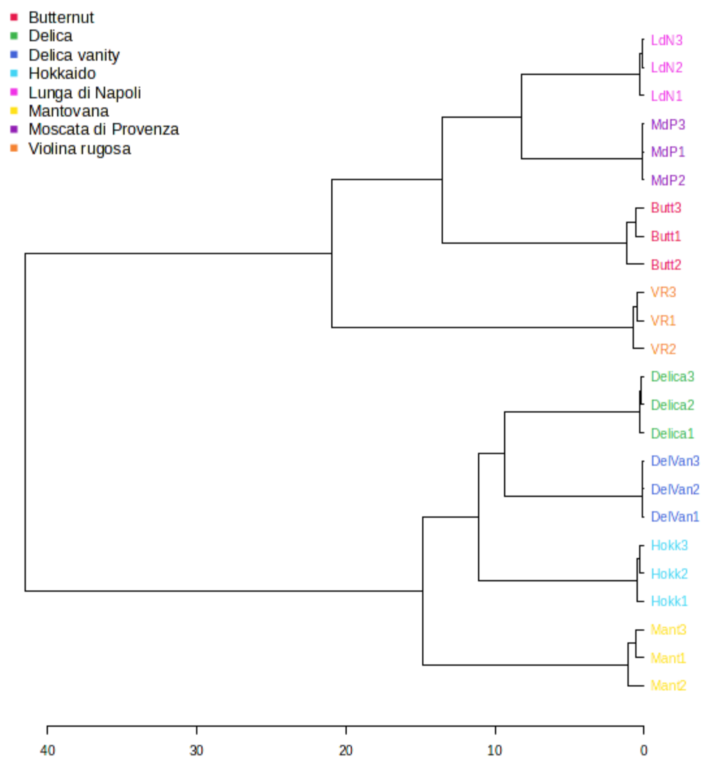


**Figure S5:** Hierarchical Clustering Dendrogram. Distance Measure: Euclidean; Clustering Algorithm: Ward.
